# Miniaturized microscope for non-invasive imaging of leukocyte-endothelial interaction in human microcirculation

**DOI:** 10.1038/s41598-023-45018-1

**Published:** 2023-10-19

**Authors:** Arutyun Bagramyan, Charles P. Lin

**Affiliations:** Wellman Center for Photomedicine and Center for Systems Biology, Massachusetts General Hospital, Harvard Medical School, Boston, MA USA

**Keywords:** Translational research, Diagnosis

## Abstract

We present a miniature oblique back-illumination microscope (mOBM) for imaging the microcirculation of human oral mucosa, enabling real-time, label-free phase contrast imaging of individual leukocytes circulating in the bloodstream, as well as their rolling and adhesion on vascular walls—the initial steps in leukocyte recruitment that is a hallmark of inflammation. Using the mOBM system, we studied the leukocyte-endothelial interactions in healthy and locally inflamed tissue and observed drastic changes in leukocyte movement (velocity and displacement profile). Our findings suggest that real-time imaging of leukocyte dynamics can provide new diagnostic insights (assessment of inflammation, temporal progression of disease, evaluation of therapeutic response, etc.) that are not available using conventional static parameters such as cell number and morphology.

## Introduction

The ability of leukocytes to traffic to various organs and tissues is a fundamental requirement for proper immune function^1^. Leukocyte recruitment is a multi-step process initiated by the slowdown of circulating leukocytes that tether and roll on the endothelial surface, followed by their firm adhesion and extravasation into tissue^2^. Though well characterized in animal models using intravital microscopy^3–5^, the rolling and adhesion events (collectively known as leukocyte-endothelial interaction, or LEI^6,7^) have rarely been observed in humans^8–10^. Conceptually, imaging cell motion as a potential source of diagnostic information has yet to be explored in clinics, as traditional histopathology has relied on the static examination of biopsied samples. LEI is reported to be significantly increased in the sublingual microvasculature of patients with systemic inflammation induced by cardiac interventions^9,10^, liver surgery^11^, and sepsis^12^. However, assessing LEI in clinical settings has been challenging due to the lack of proper detection and analytical tools. Individual leukocytes are not resolved using existing clinical instruments such as CytoCam and MicroScan^13^; instead, their presence is inferred from the gaps or voids in the blood vessels otherwise filled with red blood cells. The suboptimal image quality is further compounded by severe motion and pressure artifacts and a lack of proper analytical tools to quantify leukocyte motion^14^. Reflectance confocal microscopy (RCM) provides high-resolution cellular imaging and has been successfully used in dermatology clinics^15^. However, RCM requires bulky laser scanning systems with a limited frame rate^16^. Nonlinear optical techniques such as third-harmonic generation (THG) microscopy^17^ and two-photon-induced UV autofluorescence imaging^18^ can also provide label-free imaging of leukocytes, but these techniques entail complex laser systems and scanning platforms with no clear path for translation to the bedside.

**Figure 1:**
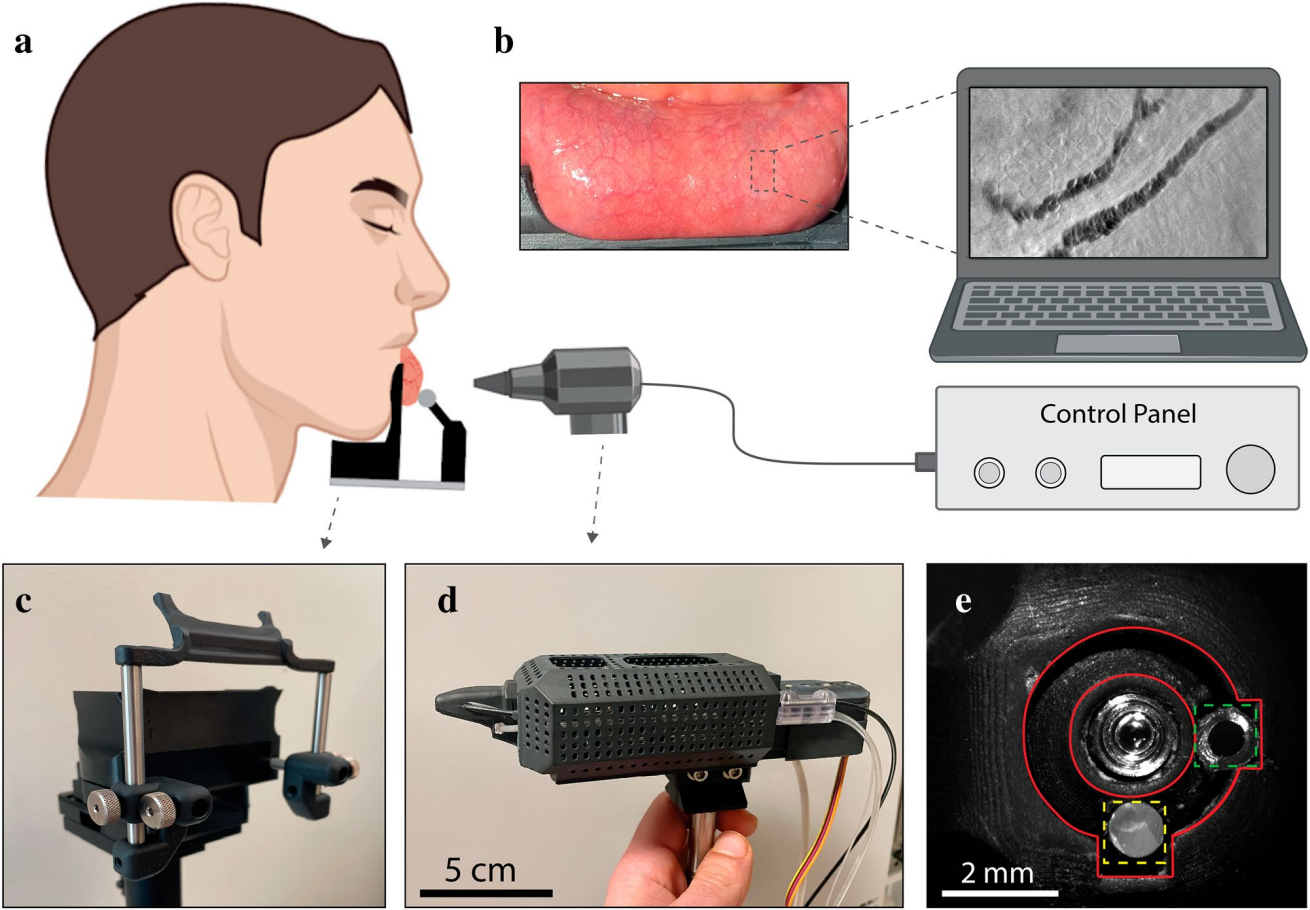
System for in vivo imaging of human oral mucosa. (**a**) Schematics of the set-up. (**b**) Exposed oral mucosa tissue of a healthy human subject. The microvasculature is shallow and easy to access with our imaging instrument presented in (**d**). (**c**) Oral mucosa apparatus system. (**d**) The developed mOBM. (**e**) The imaging tip consists of a GRIN lens (center), optical fiber (yellow dotted square), immersion medium tube (green dotted square), and the vacuum cavity (area between red circles).

**Figure 2:**
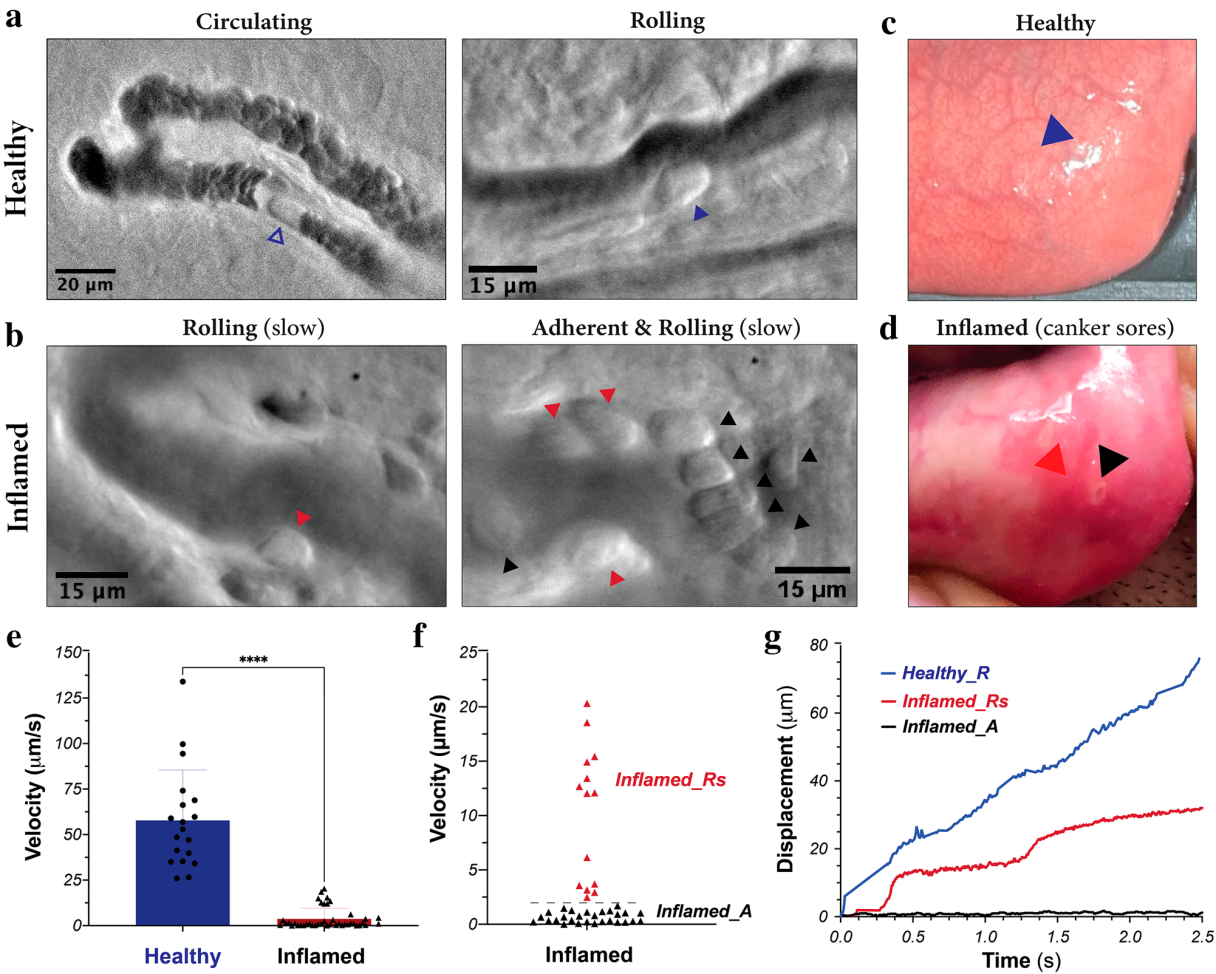
Imaging and analysis of leukocyte dynamics in the microvasculature of human oral mucosa. **(a)** Healthy condition. *Left:* A circulating leukocyte (open blue arrowhead); single frame from Supplementary Video 1. *Right:* A rolling leukocyte (blue arrowhead); averaged image of five single (consecutive) frames from Supplementary Video 2. (**b)** Inflamed condition. *Left:* A slow-rolling leukocyte (red arrowhead). *Right:* Slow-rolling and adherent leukocytes (red and black arrowheads, respectively) from Supplementary Video 3. (**c, d)** Healthy and inflamed oral mucosa tissue. Blood flow smoothing was applied on rolling and adherent cell videos (see *Methods*). (**e–g**) Velocity and displacement profiles of individual leukocytes. In (**e**), we have used unpaired t-test (blue column, n = 18; Red column, n = 39). P-value < 0.0001. *Healthy_R*: Healthy tissue, rolling cell;_*Inflamed_Rs*: Inflamed tissue, slow rolling cell; *Inflamed_A*: Inflamed tissue, adherent cell. *Note: Images of the inflamed tissue were obtained with an earlier version of the instrument (Supplementary Figure 1). Data acquisition parameters and image processing pipelines are detailed in *Methods.*

To address these limitations, we developed a miniaturized oblique back-illumination microscope (mOBM) for non-invasive imaging of the microvasculature of human oral mucosa (Fig. 1). The imaging tip of mOBM (Fig. 1d, e) consists of a 1 mm diameter aberration-corrected gradient index (GRIN) objective lens with a numerical aperture (NA) of 0.75 and a large core (Ø 1 mm) multimode optical fiber coupled to a green LED (light emitting diode) light source. The output end of the fiber is positioned such that photons enter the tissue from one side of the GRIN lens (Fig. 1e). Injected photons undergo multiple scattering events in deep tissue layers, and a fraction of the photons are collected by the GRIN lens, providing the back-illumination at an oblique angle due to the offset geometry of the optical fiber^19^. The asymmetric oblique back illumination generates phase-gradient contrast (PGC) images that resemble differential interference contrast (DIC)^20^ or differential phase contrast (DPC)^21^ microscopy images with a crucial difference. Unlike DIC or DPC, OBM is designed to work with intact, thick tissue^19^ and is therefore compatible with in vivo imaging. The use of the green LED serves to amplify contrast through hemoglobin absorption^22,23^, which makes white blood cells stand out against the darker red blood cells (Fig. 2a, left).

## Methods

### Key optomechanical features of the mOBM system

The optical design of mOBM was accomplished using the Zemax raytracing software (Supplementary Figure 2). It consists of a 1 mm diameter gradient index (GRIN) objective lens assembly, relay optics, and a CMOS camera. The imaging GRIN lens assembly (Grintech, *GT-MO-080-032-ACR-VISNIR-08-20*) is aberration-corrected and has a high NA of ≈0.75 (in water). The relay optics consists of a second GRIN lens (Edmund Optics, *#64-519*) with a ≈ 1/4 pitch and NA≈0.52 together with an achromat doublet lens (Edmund Optics, *#49-772*) with an effective focal length of 19.1 mm that projects the magnified image onto the CMOS sensor (Basler, *daA1920-160um*). Oblique illumination is provided by a 530 nm LED light source (Thorlabs, *M530F2*) coupled to a 1 mm diameter large core optical fiber, whose output (≈ 30 mW) end is positioned on one side of the GRIN objective lens assembly (Fig. 1e). The assembled mOBM instrument (Fig. 1d) is designed to detect multiply scattered photons in a non-confocal manner, generating phase-gradient contrast (PGC) images that reveal fine morphological details (e.g., cell boundaries, intracellular granules, etc.). In addition to the PGC, our instrument also benefits from absorption-contrast generated by using the 530 nm wavelength that is strongly absorbed by hemoglobin (in red blood cells) and not by leukocytes^22,23^. An important element is the imaging tip (Fig. 1e), which consists of four channels: (1) a central channel containing the miniaturized GRIN objective lens. (2) An illumination channel containing the large core multi-mode fiber. (3) An irrigation channel to keep oral mucosa tissue moist and to maintain the immersion medium in front of the objective lens. (4) A ring-shaped vacuum cavity surrounds the central channel to stabilize the imaging area. The magnification of our instrument was ≈ 25, which resulted in ≈ 7.2 pixels/µm (without binning) and ≈ 3.6 pixels/µm (with 2 × 2 binning) ratios. The field-of-view of our system was ≈ 150 × 200 µm and the working distance of the objective lens was adjusted (20–100 µm) by means of motorized displacement of the camera in the direction of the optical axis. SolidWorks software was used to design mOBM’s mechanical housing, the imaging tip, and the oral mucosa apparatus (Fig. 1c). Parts were printed using a 3D laser printer (Formlabs 3B). All parts in contact with the human tissue were printed with sterilizable, biocompatible materials (Formlabs, *RS-F2-BMCL-01, RS-F2-BMBL-01*).

### Tissue stabilizer

The universal oral mucosa apparatus (Fig. 1c) was used to gently hold the subject’s lower lip tissue and expose buccal microvasculature (Fig. 1b) without impeding the blood flow. The apparatus also stabilized the tissue for the duration of the imaging session (≈ 20–60 min). A motorized XYZ stage was used to position the imaging tip of the mOBM and locate the microvasculature of interest.

### Imaging of healthy participant

The imaging session and data collection started after the subject had signed the IRB-approved consent form. The subject was first seated in front of the imaging system, and the head was gently stabilized using soft tissue straps. The lower lip was then exposed and stabilized using the oral mucosa apparatus (Fig. 1c). There were no additional requirements from the subject other than remaining in a seating position for the duration of the imaging. The imaging session was initiated by the operator who positioned the imaging tip over the region of interest (ROI) using a motorized XYZ actuator with micrometer resolution. It typically took a few minutes to locate a suitable vessel and acquire a video. The imaging session usually involved capturing multiple videos and lasted between 20 and 60 min. Data was collected from a single subject, in 3 imaging sessions spread over a 2-month period. At the 2nd imaging session, a canker sore (localized inflammation) was noted on one side of the lip.

### Image acquisition parameters

For the circulating (Fig. 2a, left) and rolling leukocytes (Fig. 2a (middle) and Fig. 2b), the acquisition framerate of the camera was fixed at 200 fps and the exposure time was ≈0.5 ms. If necessary, 2 × 2 binning was used to increase the signal–noise ratio.

### Image processing pipeline

Image processing was performed in *ImageJ* (open-source software). The image processing pipeline for the rolling and adherent leukocytes: (1) Registration (plug-in: *Template Matching).* (2) Cropping the region of interest (ROI). (3) Blood flow smoothing (plug-in: *Kalman filter*). (4) Extraction of PGC (Image—Gaussian blurred image (sigma radius: 20–40)). (5) Average subtraction (PGC stack—averaged image) (6) Leukocyte tracking (plug-in: *TrackMate*). Leukocytes that detached from the endothelial wall after brief contact(s) were excluded from the analysis. The image processing pipeline for the circulating leukocytes: (1) Registration (plug-in: *Template Matching).* (2) Cropping the ROI. (3) Extraction of PGC (Image—Gaussian blurred image (sigma radius: 20–40)).

### Ethical approval

The experimental protocols related to human volunteers were performed in accordance with the guidelines and regulations of Massachusetts General Hospital. The study protocol (#2021P003047) was approved by the Internal Review Board (IRB) of Massachusetts General Hospital. Informed consent was obtained from all subjects participating in our study.

## Results

To validate the performance of our system, we imaged the movements of leukocytes in the microvasculature of human oral mucosa (Fig. 2a–d). When imaged at a frame rate of 200 Hz, individual blood cells in the microcirculation are clearly delineated (Fig. 2a, left; Supplementary Video 1). These cells travel at a speed of ≈1 mm/sec, and their motions are effectively “frozen” when the exposure time of individual frames is set to < 0.5 ms. In addition, rolling cells are detected in a subset of blood vessels of healthy volunteers (Fig. 2a, right; Supplementary Video 2), likely postcapillary venules based on their diameter and flow speed. The rolling cells move at a much lower velocity of ≈ 58 ± 28 µm/s and remain sharply defined even with long exposure times.

We also imaged an inflamed area caused by the presence of canker sores (Fig. 2d) and observed a drastic reduction in the average speed of leukocytes (Supplementary Video 3). Using automated frame-by-frame leukocyte tracking (see *Methods*), we obtained an average rolling velocity of 58 ± 28 µm/s in the healthy tissue, which reduced to 4 ± 6 µm/s in the inflamed tissue (Fig. 2e). Closer inspection revealed two populations (Fig. 2f), an adherent population with an average velocity ≈ 0.7 ± 0.7 µm/s and a slowly rolling population with an average velocity of 11 ± 6 µm/s. The adherent cells were restricted to the area close to the canker sores (Fig. 2d, black arrowhead), while the slowly moving leukocytes were detected both in the center and in the periphery (Fig. 2d, red arrowhead) of the inflamed area. The displacement profile showed the characteristic stop-and-go movement patterns during inflammation (Fig. 2g).

## Discussion

Non-invasive in vivo imaging of blood cell dynamics is an established method for studying immune response at the cellular level in animal models of diseases including cancer^24^, cardiovascular disease^25^, and inflammation^26^. Translating this powerful method to human imaging will enable a multitude of clinical applications such as disease diagnosis and monitoring, treatment evaluation, early detection, image-guided procedures, etc. Successful clinical translation however has been hampered by the lack of proper imaging and analytical tools. To address this need, we have built the mOBM, a compact instrument that enables non-invasive and label-free temporal recordings of circulating, rolling, and adherent leukocytes within the human buccal microvascular. Here, we describe the main design features of our system, guided by the recommendations detailed in the literature^14^, along with insights derived from the assessment of the device in human subjects.

### Choice of tissue site for imaging

We have opted to image the oral mucosa tissue due to its well-developed and shallow vascular bed, lack of skin pigmentation, and the absence of the highly scattering stratum corneum layer that degrades image quality in the skin. When selecting the specific imaging area within the oral mucosa, subject’s comfort and tissue stabilization were of utmost importance, to minimize motion artifacts and fatigue during the imaging sessions. Our investigation revealed that imaging of the inner sections of the oral mucosa, such as the sublingual area, was challenging for individuals engaged in extended imaging sessions (> 10 min), due to the open-mouth position, which resulted in the subject’s fatigue. The conventionally considered inner cheek tissue was a potential alternative that was tested but not pursued due to the challenges in adequately exposing and stabilizing the microvasculature of interest. Ultimately, we have chosen to capture images in the lower lip, which is in the proximal oral area that is easy to access. We have designed a custom apparatus to expose the inner microvasculature of the lip and sustain a stable position throughout the imaging, all while minimizing the discomfort experienced by the subjects.

### Oral tissue stabilization apparatus

Motion and pressure artifacts pose major challenges in high-resolution imaging of blood cells. With current clinical handheld vital microscopes, the operator is required to subjectively gauge the force to achieve stabilization of the imaging region while concurrently avoiding hemodynamic disruption, which is a difficult balancing act. Vacuum-based stabilization techniques are similarly prone to perturbing flow dynamics in small vessels and capillaries. Overall, the difficulty posed by conventional methods originates from the attempt to use the instrument's tip as the primary means of stabilizing the tissue. In our design, a dedicated mechanical apparatus was conceived to gently expose the lower lips’ microvasculature (Fig. 1b) and stabilize the tissue of interest without affecting the blood flow dynamics. To do so, the contact points between the tissue and apparatus were deliberately situated at a considerable distance from the actual imaging area (Fig. 1b). We believe this approach offers a lower risk of disturbing the blood flow compared to the existing method, which involves applying pressure directly to the microvasculature of interest using the instrument's tip. Furthermore, to maximize the subject’s comfort and avoid high-pressure points, careful consideration went into the mechanical design of the apparatus that was meticulously aligned with the natural contours of the lip, assuring an equal distribution of forces throughout the contact points. With the tissue effectively stabilized, a micron-resolution actuator system was used to guide the tip of mOBM across the lip's surface in search of vessels of interest. The vacuum channel built into the imaging tip was only used sparingly as a secondary means of tissue stabilization when needed (about ≈5% of the time).

### Instrument design

We have opted to use an oblique back-illumination imaging modality that allows visualization of fine morphological features of blood cells (e.g., cell boundaries and intracellular granules) by means of phase contrast signal^19^. The oblique back-illumination approach also benefits from a good signal–noise ratio, low light input requirement, and simple components that can be miniaturized. After weighing design considerations such as image quality, device portability, cost efficiency, and ease of use, we have settled on the mOBM with miniaturized optical components such as the GRIN and aspheric lenses (Supplementary Figure 2). As an objective lens, we used an aberration-corrected GRIN lens assembly with NA = 0.75 that provides high image quality with a reasonable field of view (FOV, about 200 µm). In addition to the GRIN lens, an immersion medium channel, a vacuum cavity, and an optical fiber cavity are all integrated into the imaging tip (Fig. 1e). The compact design of the tip proved crucial in facilitating access and easy navigation throughout the lip’s exposed microvasculature. To capture the back-scattered photos we have used a compact CMOS detector capable of high acquisition rates (up to 1000 fps with ROI cropping) to image rapidly moving cells in the bloodstream (Fig. 2a, left).

### Initial human observations

To validate our device, we first show the ability of mOBM to visualize rapidly moving blood cells in the human oral mucosa microcirculation. By employing a high acquisition rate of 200 fps, we were able to efficiently capture the movement of circulating cells (both erythrocytes and leukocytes) in the bloodstream (Supplementary Video 1) across multiple video frames. Additionally, the camera's short integration times (≈0.5 ms) played a crucial role in reducing the blurring caused by the rapid motion of cells. The presence of a characteristic gap in blood plasma in front of the moving leukocyte (Fig. 2a, left) arises because the flow velocity of leukocytes tends to be slightly lower relative to red blood cells. The phase gradient contrast generated by mOBM enabled visualization of cell boundaries, improving the efficacy of the detection of leukocytes distinct from plasma gaps (Fig. 2a, left).

Next, we demonstrated the ability of the mOBM to visualize the interaction between leukocytes and the blood vessel wall in healthy and inflamed human tissues. In the healthy condition, leukocytes exhibited individual rolling behaviors, occasionally coming into contact with other rolling or circulating leukocytes. The average speed of these leukocytes was ≈58 ± 28 µm/s, and the displacement profile showed a monotonic increase in distance with time (Fig. 2g, blue curve). However, within the same subject, these movements differed significantly in the presence of inflammation. At the site of infection, two distinct groups of leukocytes were observed: one group exhibited slow rolling velocities (11 ± 6 µm/s) with a characteristic stop-and-go movement profile (Fig. 2g, red curve), while the other group was essentially stationary (0.7 ± 0.7 µm/s) and formed cell clusters (Fig. 2b, middle). The phase-contrast signal of mOBM played a crucial role here, enabling the distinction of individual leukocytes within the cluster. Achieving this level of detection and quantification would have been impossible with existing medical devices^13^, where white blood cells appear transparent and consequently undetectable either individually or when clustered.

Notably, the leukocyte rolling velocity in the periphery of the canker sores was also significantly affected (Fig. 2d, red arrow), but the rolling cells did not come to a complete stop. This observation suggests that endothelial cells in the peripheral areas of inflammation express selectins, while in the center of inflamed tissue, they additionally express high levels of cell adhesion molecules such as VCAM-1 and ICAM-1^27^.

Overall, the ability to monitor LEIs within inflamed tissue highlights the potential of mOBM in diagnosing the degree of inflammation, tracing its distribution within the tissue, and gaining deeper insights into the immune cell response against different types of pathogens. We are particularly interested in using mOBM to assess LEIs in systemic inflammation such as sepsis^12^, where imaging the vasculature of the oral mucosa can provide valuable insights into the systemic immune status. Given the findings outlined in our study, we anticipate mOBM will enhance the leukocyte detection and movement analysis compared to current clinical devices, providing novel perspectives on the body's immune response for potential diagnosis and treatment.

For future work, the instrument can be further miniaturized by replacing the current focusing mechanism (a translational stage) with an electrically tunable lens^28^. In addition, the high-speed CMOS sensor (frame rate up to 1000 Hz) can be replaced by a standard (30 Hz) video camera that will still be able to image rolling and adherent cells, but the flowing cells will not be resolved at this frame rate. The high-speed imaging capability offers the tantalizing possibility of performing non-invasive white blood cell count by resolving individual circulating cells and flagging leukocytes "on the fly" with the help of machine learning, a subject of active pursuit in our laboratory and others^29^. Because of its compact size, low cost, and simple construction, we expect to place the mOBM instrument in several clinics to begin testing the diagnostic utility in critically ill patients and preterm infants at high risk of infection and sepsis. We further envision the instrument to find use in resource-poor settings that lack the expertise and infrastructure to draw blood for standard laboratory analysis.

### Supplementary Information

Supplementary Information 1.

Supplementary Figures.

Supplementary Video 1.

Supplementary Video 2.

Supplementary Video 3.

## Data Availability

The data that support the findings of this study are available from the corresponding author upon reasonable request.
