# Supplementary material for: Miniaturized microscope for non-invasive imaging of leukocyte-endothelial interaction in human microcirculation
Source: Sci Rep. 2023 Oct 19;13:17881. doi: 10.1038/s41598-023-45018-1 (PMC10587353; doi:10.1038/s41598-023-45018-1)
Supplement: Supplementary file 1 — Supplementary Information 1. [file 41598_2023_45018_MOESM1_ESM.docx]

**Video Legends:**

**Supplementary Video 1:** Circulating leukocytes in the oral mucosa microvasculature of a healthy volunteer. Acquisition frame rate: 200fps.

**Supplementary Video 2:** Rolling leukocytes in healthy oral mucosa tissue. Acquisition frame rate: 200fps.

**Supplementary Video 3:** Adherent and slowly rolling leukocytes in inflamed oral mucosa tissue. Acquisition frame rate: 200fps.
