# Supplementary material for: Miniaturized microscope for non-invasive imaging of leukocyte-endothelial interaction in human microcirculation
Source: Sci Rep. 2023 Oct 19;13:17881. doi: 10.1038/s41598-023-45018-1 (PMC10587353; doi:10.1038/s41598-023-45018-1)
Supplement: Supplementary file 2 — Supplementary Figures. [file 41598_2023_45018_MOESM2_ESM.docx]

**Supplementary Figures:**

**
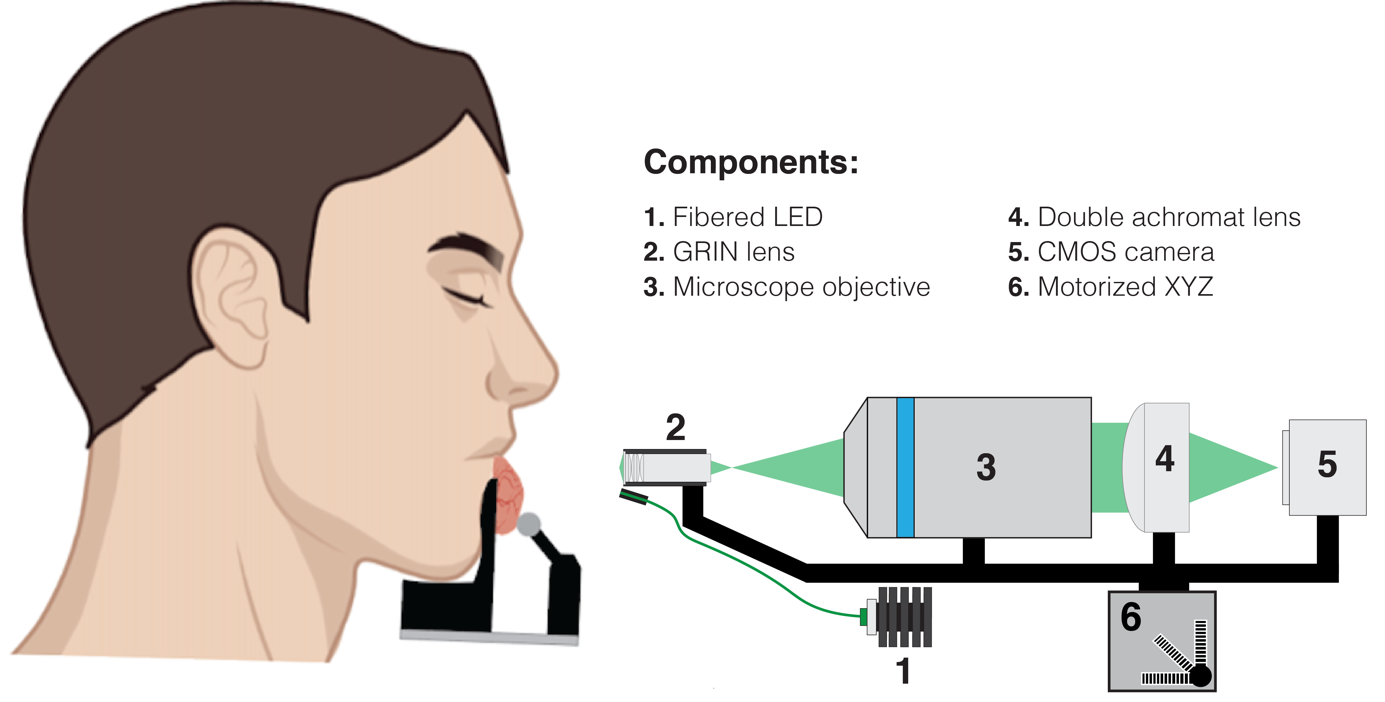
Supplementary Figure 1 |** An earlier version of the mOBM system for oral mucosa imaging in human subjects uses a microscope objective (Mitutoyo, *378-804-3*) that was later replaced with a GRIN lens (Supplementary Figure 2, component #2).

**
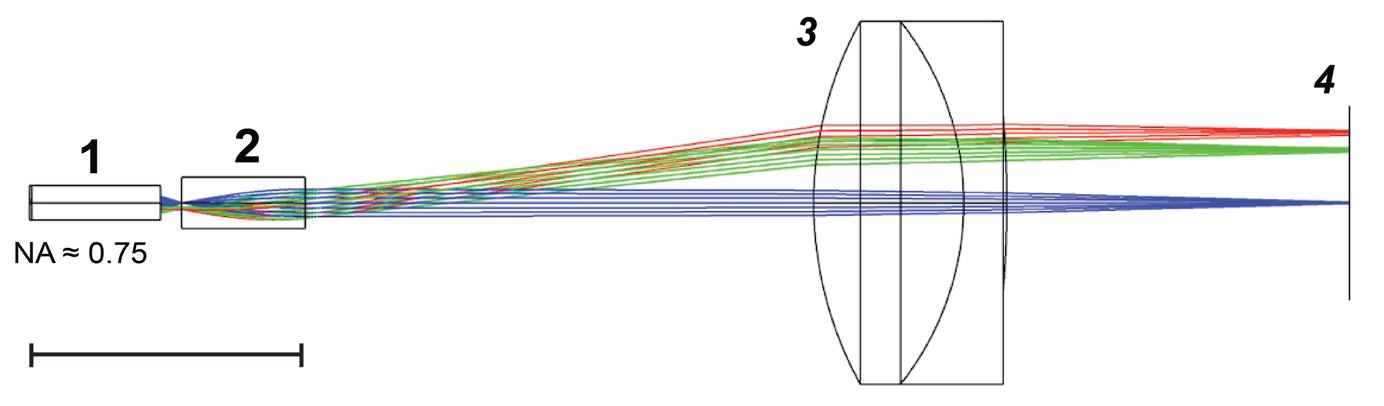
**

**Supplementary Figure 2** **|** Optical simulation (Zemax) of the imaging pathway of the miniaturized instrument (Fig. 1d). Components: 1. Miniaturized aberration-corrected GRIN lens assembly, NA ≈ 0.75 (in water); 2. Standard GRIN lens, N=0.52; 3. Achromat doublet lens, EFL = 19.1 mm 4. Monochrome CMOS camera.
